# Supplementary material for: Multi-scale model suggests the trade-off between protein and ATP demand as a driver of metabolic changes during yeast replicative ageing
Source: PLoS Comput Biol. 2022 Jul 7;18(7):e1010261. doi: 10.1371/journal.pcbi.1010261 (PMC9295998; doi:10.1371/journal.pcbi.1010261)
Supplement: S1 Text — (PDF) [file pcbi.1010261.s002.pdf]

Multi-scale model suggests the trade-off between protein and ATP demand as a driver of metabolic changes during yeast replicative ageing
Supplementary text 1: Model details

Barbara Schnitzer [*1,2], Linnea Österberg [*3], Iro Skopa [1,2], Marija Cvijovic [1,2]

June 8, 2022

[*] Authors contributed equally
[1] Department of Mathematical Sciences, Chalmers University of Technology, Gothenburg, Sweden
[2] Department of Mathematical Sciences, University of Gothenburg, Gothenburg, Sweden
[3] Department of Biology and Biological Engineering, Chalmers University of Technology, Gothenburg, Sweden

Contents

1 Boolean model of cellular signalling **2**
1.1 Theoretical background 2
1.2 Addition of oxidative stress signalling 2

2 Enzyme-constrained flux balance analysis of the metabolic network **2**
2.1 Theoretical background 2
2.2 Addition of damage producing reactions 3

3 Dynamical model of growth, cell division and damage accumulation **4**

1 Boolean model of cellular signalling

1.1 Theoretical background

Vector-based Boolean models are powerful tool to understand the topology of a network. They are generally based on logical arguments and moreover parameter-free. Given a network, each of its N components is represented by a k-dimensional vector of binary states $\mathbf{p}_i \in \{0,1\}^k$, $i = 1..N$, that represent chosen properties. The state of each component i can be altered based on all other states by Boolean rules or functions $\mathcal{B}$: $(\mathbf{p}_1, \mathbf{p}_2, .., \mathbf{p}_N) \rightarrow \mathbf{p}_i$. In each iteration all Boolean functions $\mathcal{B}_j$, $j = 1..M$, are applied synchronously to the states to generate updated states for the next step. Eventually, the system can end up in a logical steady state where the states of all components remain constant.

This formalism can be used to understand signalling events in cells in order to find the eventual activation of transcription factors. Here, we used a vector-based Boolean model of the nutrient signalling pathways Snf1, Tor and PKA that was previously published [1] and extended it further with the oxidative stress signalling pathways Yap1 and Sln1, as well as crosstalk to Msn2/4 that is also part of the nutrient signalling pathway PKA (Fig 1A). Each component in the network is represented by the properties presence, phosphorylation, oxidation and specific activity ($k = 4$). The Boolean rules are based on an extensive literature review to describe the signalling events, and correspond to simple if-statements, such as: IF protein X is present and phosphorylated AND protein Y is present and oxidised then protein Z gets phosphorylated. The activity property is especially important in our setting and is interpreted as being in the nucleus and interacting with the DNA, which can lead to expression or repression of genes. In particular, one can therefore investigate how perturbed input signals regarding nutrient availability and stress can influence the resulting transcription factor activity. Moreover, Boolean models generally allow to find logical gaps in the network [2, 3].

1.2 Addition of oxidative stress signalling

In total, we added 9 new components and 13 new rules, including 1 crosstalk reaction, to the existing model of nutrient signalling [1] to account for oxidative stress signalling by the Yap1 and the Sln1 pathway.

Yap1
Yap1 mediates ROS stress signalling by sensing of H_2O_2 mediated trough the peroxidin Gpx3[4, 5]. In the presence of H_2O_2 Gpx3 together with Ybp1 facilitates the formation of active Yap1 [4, 5] that will accumulate in the nucleus where it induces it's gene targets including SOD1, GSH1, GPX2, TRX2 and TSA1 [6, 7]. The pathway returns to it's reduced state when reduced by thioredoxin, which is also a target gene of Yap1 [8].

Sln1
The Sln1 pathway is associated with osmoregulatory response, where Sln1 regulates Ypd1 phosphorylation. Ypd1 akts on the Ssk1 and on the transcription factor Skn7 [9, 10]. The role of this pathway in ROS regulation is not elucidated, but there are increasing reports of it's association to oxidative stress response where Skn7 plays a role, alone and in connection with Yap1 [9, 10].

Crosstalk to nutrient signalling
Msn2 and Msn4 are central stress regulators, targeting an number of oxidative response genes. The activation in respons to oxidative stress is mediated trough the thioredoxins Trx1 and Trx2[11]. Knockouts of Msn2 or Msn4 exhibit hypersensitivity to H_2O_2 and the response is only partially overlapping with that of Yap1 and Skn7 [12].

2 Enzyme-constrained flux balance analysis of the metabolic network

2.1 Theoretical background

Generally, in flux balance analysis (FBA) [13–15] chemical reactions in the network are represented by a stoichiometric matrix S. Assuming that each component can only be used as much as it is produced, the

system is naturally constraint by this mass balance requirement. Mathematically, the fluxes $\mathbf{v}$ through the network have to satisfy $S\mathbf{v} = 0$. Given an objective function, that can be an individual flux or a combination of several fluxes, all fluxes can be optimised accordingly. Biologically, examples for objective functions are maximal growth, minimal nutrient uptake or maximal growth yield. The optimal solution can be found by solving the linear program in (1).

$$\begin{aligned} \text{optimise} \quad & z = \mathbf{c}^T \mathbf{v} \\ s.t. \quad & S\,\mathbf{v} = \mathbf{0} \\ & \mathbf{v}_{min} \leq \mathbf{v} \leq \mathbf{v}_{max}, \end{aligned} \tag{1}$$

with $\mathbf{c}$ defining the coefficients of the fluxes in the objective function. Furthermore, $\mathbf{v}_{min}$ and $\mathbf{v}_{max}$ are general lower and upper bounds on the fluxes.

Enzyme-constrained FBA (ecFBA) [16, 17] is an extension of the traditional FBA, incorporating enzymes as components that are required for catalysing certain reactions. Each enzyme e_i that is used is drawn from an enzyme pool e_{pool} and is consumed in one or more reactions with a stoichiometric coefficient inversely proportional to its respective turnover number k_{cat}. The enzyme pool is itself restricted by the total amount of proteins P_{tot} in the cell. The new additional constraints in the optimisation problem are stated in (2).

$$\begin{aligned} s.t. \quad & -\sum_j \frac{n^{ij}}{k_{cat}^{ij}} v_j + e_i = 0, \qquad \forall i \\ & -\sum_i MW_i e_i + e_{pool} = 0 \\ & \mathbf{e}_{min} \leq \mathbf{e} \leq \mathbf{e}_{max} \\ & 0 \leq e_{pool} \leq \sigma f P_{tot}, \end{aligned} \tag{2}$$

with n^{ij} being the number of enzymes i that are needed to catalyse reaction j. In most cases n^{ij} equals 0 or 1, but can in exceptional cases of enzyme complexes be higher. Further, MW_i are the molecular weights of the enzymes, f corresponds to the fraction of the total protein mass covered by the enzymes in the model and σ to the saturation factor of the enzymes. Similar to before, $\mathbf{e}_{min}$ and $\mathbf{e}_{max}$ are general lower and upper bounds on the enzyme usages. Typically, each optimisation is followed up by a second optimisation that picks the solution with a minimal sum of all fluxes and enzyme usages (parsimonious FBA). EcFBA has been shown to improve the predictive power in comparison to the traditional FBA [1, 17].

Note that fluxes typically have the unit $[mmol(gDWh)^{-1}]$ or $[h^{-1}]$, while enzyme usages are measured in $[mmol(gDW)^{-1}]$ and protein content in $[g(gDW)^{-1}]$.

2.2 Addition of damage producing reactions

In this work, we make use of a previously published ecFBA model of the central carbon metabolism [1, 16] and incorporated new chemical reactions that produce reactive oxygen (ROS) and nitrogen species (RNS) (Fig 1B).

The new reactions are based on the fact that while cells produce energy in the mitochondria about 0.2-2% electron leak from the electron transport chain (ETC) [18]. Complex 3 in the ETC can be responsible for some of those electrons, while most of them escape from complex 1 [18–20]. The major downstream ROS and RNS reactions that are caused by the free electrons are summarised in the following according to [21–27]. When electrons react with oxygen (O_2) the negatively charged superoxide (O_2^-) is produced. O_2^- can be transformed to H_2O_2 via superoxide oxidoreductase (SOD1, SOD2). H_2O_2 can be transformed back to water by glutathione peroxidase (GPX1-3). In that reaction glutathione disulfide gets glutathione. To transform back glutathione to glutathione disulfide the enzyme glutathione oxidoreductase (GLR1) is needed. Similar reactions happen for thioredoxin instead of glutathione, using thioredoxin peroxidase (TRX1-3) and reductase (TRR1-2). In addition, H_2O_2 transforms to $OH^\cdot$ via Fenton- and Haber-Weiss reactions with iron cations as mediators. $OH^\cdot$ can also be indirectly produced by $OONO^-$, encompassing several reactions that besides

others convert the nitric oxide radical $NO^{\cdot}$ to $NO_2^{\cdot}$. In this simplified pathway the major cause of damage is the hydroxyl radical ($OH^{\cdot}$) that can oxidise proteins and make them dysfunctional.
In addition, we introduced a non-growth associated ATP cost (NGAM) to the model, $NGAM(t) = \frac{D(t)}{P(t)+D(t)} \cdot NGAM_{max}$., with the $NGAM_{max}$ as in [28].
In total, it resulted in 52 new reactions and 41 new components including 13 new enzymes in the ecFBA model compared to [1]. Necessary k_{cat} values were adopted from the consensus yeast metabolic model [28].

3 Dynamical model of growth, cell division and damage accumulation

To describe the protein damage accumulation over time, we make use of an ordinary differential equation (ODE) model, that is based on three forces: damage formation, damage repair and cell growth. The biomass of a cell M $[gDW]$ follows a simple linear ODE with a time-dependent growth factor $g(t)$.

$$\frac{\mathrm{d}M(t)}{\mathrm{d}t} = g(t)M(t). \tag{3}$$

Further, the fractional intact (P) and damaged (D) protein content $[g(gDW)^{-1}]$ are described by two coupled ODEs. Intact proteins get damaged at a rate $f(t)$ and damaged proteins are repaired at a rate $r(t)$, such that

$$\frac{\mathrm{d}P(t)}{\mathrm{d}t} = -f(t)P(t) + r(t)D(t) \tag{4}$$

$$\frac{\mathrm{d}D(t)}{\mathrm{d}t} = +f(t)P(t) - r(t)D(t). \tag{5}$$

We assume the total protein fraction to be constant $P(t) + D(t) = const$, however the composition of intact and damaged proteins changes over time.
For constant rate parameters $g(t) = g$, $f(t) = f$ and $r(t) = r$ the solutions to Eq (3)-(4) can easily be obtained by calculating eigenvalues and eigenvectors.

$$M(t) = M(0) \cdot e^{gt} \tag{6}$$

$$P(t) = \frac{1}{f+r} \cdot \left[r(P(0) + D(0)) - (D(0)r - P(0)f)e^{-(f+r)t} \right] \tag{7}$$

$$D(t) = \frac{1}{f+r} \cdot \left[f(P(0) + D(0)) + (D(0)r - P(0)f)e^{-(f+r)t} \right]. \tag{8}$$

We incorporate cell division as a discrete instantaneous event in the model. Let $s \in [0.5, 1]$ denote the size (= mass) proportion of the mother cell at cell division. Then, as soon as enough biomass has been produced, $M(t_d) = s^{-1}M(0)$, the cell can divide into a mother cell and a daughter cell of sizes

**mother**	**daughter**	(9)
$M \leftarrow sM(t_d) = M(0)$	$M \leftarrow (1-s)M(t_d) = (1-s)s^{-1}M(0).$	

At the same time, the total fractional protein content in both compartments remains constant. Without damage retention mechanisms, we assume that also P and D individually remain constant across the compartments. Increasing the retention factor $re \in [0, 1]$ accounts for the asymmetric distribution of damage at cell division [29, 30], resulting in a higher fraction of damaged proteins in the mother cell compartment and a lower fraction of damaged proteins in the daughter cell compartment. To ensure that the masses in both compartments are conserved, the fraction of intact proteins is at the same time decreased or increased respectively in mother and daughter. Consequently, if at cell division the content is $P(t_d)$ and $D(t_d)$, the variables are updated according to

**mother**	**daughter**
$P \leftarrow (1 - re)P(t_d)$	$P \leftarrow (1 + re)P(t_d)$
$D \leftarrow (1 + re)D(t_d)$	$D \leftarrow (1 - re)D(t_d).$

(10)

References

1. Österberg, L. *et al.* A novel yeast hybrid modeling framework integrating Boolean and enzyme-constrained networks enables exploration of the interplay between signaling and metabolism. *PLOS Computational Biology* **17** (ed Csikász-Nagy, A.) e1008891 (Apr. 2021).
2. Lubitz, T. *et al.* Network reconstruction and validation of the Snf1/AMPK pathway in baker's yeast based on a comprehensive literature review. *npj Systems Biology and Applications* **1.** Number: 1 Publisher: Nature Publishing Group, 1–10 (Oct. 2015).
3. Welkenhuysen, N., Schnitzer, B., Österberg, L. & Cvijovic, M. Robustness of Nutrient Signaling Is Maintained by Interconnectivity Between Signal Transduction Pathways. *Frontiers in Physiology* **9,** 1964 (Jan. 2019).
4. Delaunay, A., Pflieger, D., Barrault, M.-B., Vinh, J. & Toledano, M. B. A Thiol Peroxidase Is an H2O2 Receptor and Redox-Transducer in Gene Activation. *Cell* **111,** 471–481 (Nov. 2002).
5. Veal, E. A., Ross, S. J., Malakasi, P., Peacock, E. & Morgan, B. A. Ybp1 Is Required for the Hydrogen Peroxide-induced Oxidation of the Yap1 Transcription Factor. *Journal of Biological Chemistry* **278,** 30896–30904 (Aug. 2003).
6. Grant, C. M., Collinson, L. P., Roe, J.-H. & Dawes, I. W. Yeast glutathione reductase is required for protection against oxidative stress and is a target gene for yAP-1 transcriptional regulation. *Molecular Microbiology* **21,** 171–179 (July 1996).
7. Dumond, H., Danielou, N., Pinto, M. & Bolotin-Fukuhara, M. A large-scale study of Yap1p-dependent genes in normal aerobic and H2O2-stress conditions: the role of Yap1p in cell proliferation control in yeast. *Mol Microbiol* **36,** 830–845 (May 2000).
8. Izawa, S. *et al.* Thioredoxin Deficiency Causes the Constitutive Activation of Yap1, an AP-1-like Transcription Factor in Saccharomyces cerevisiae. *Journal of Biological Chemistry* **274,** 28459–28465 (Oct. 1999).
9. Singh, K. K. The Saccharomyces cerevisiae sln1p-ssk1p two-component system mediates response to oxidative stress and in an oxidant-specific fashion. *Free Radical Biology and Medicine* **29,** 1043–1050 (Nov. 2000).
10. He, X.-J., Mulford, K. E. & Fassler, J. S. Oxidative Stress Function of the *Saccharomyces cerevisiae* Skn7 Receiver Domain. *Eukaryot Cell* **8,** 768–778 (May 2009).
11. Boisnard, S. *et al.* H2O2 Activates the Nuclear Localization of Msn2 and Maf1 through Thioredoxins in Saccharomyces cerevisiae. *Eukaryot Cell* **8,** 1429–1438 (Sept. 2009).
12. Hasan, R. *et al.* The control of the yeast H2O2 response by the Msn2/4 transcription factors. *Mol Microbiol* **45,** 233–241 (July 2002).
13. Varma, A. & Palsson, B. O. Stoichiometric flux balance models quantitatively predict growth and metabolic by-product secretion in wild-type Escherichia coli W3110. *Appl Environ Microbiol* **60,** 3724–3731 (Oct. 1994).
14. Price, N. D., Reed, J. L. & Palsson, B. O. Genome-scale models of microbial cells: evaluating the consequences of constraints. *Nat Rev Microbiol* **2,** 886–897 (Nov. 2004).
15. Orth, J. D., Thiele, I. & Palsson, B. Ø. What is flux balance analysis? *Nat Biotechnol* **28,** 245–248 (Mar. 2010).
16. Nilsson, A. & Nielsen, J. Metabolic Trade-offs in Yeast are Caused by F1F0-ATP synthase. *Scientific Reports* **6,** 22264 (Mar. 2016).
17. Sánchez, B. J. *et al.* Improving the phenotype predictions of a yeast genome-scale metabolic model by incorporating enzymatic constraints. *Molecular Systems Biology* **13,** 935 (Aug. 2017).
18. Zhao, R.-Z., Jiang, S., Zhang, L. & Yu, Z.-B. Mitochondrial electron transport chain, ROS generation and uncoupling. *Int J Mol Med* (May 2019).
19. Nickel, A., Kohlhaas, M. & Maack, C. Mitochondrial reactive oxygen species production and elimination. *Journal of Molecular and Cellular Cardiology* **73,** 26–33 (Aug. 2014).

20. Fang, J., Wong, H.-S. & Brand, M. D. Production of superoxide and hydrogen peroxide in the mitochondrial matrix is dominated by site IQ of complex I in diverse cell lines. *Redox Biology* **37,** 101722 (Oct. 2020).

21. Temple, M. D., Perrone, G. G. & Dawes, I. W. Complex cellular responses to reactive oxygen species. *Trends in Cell Biology* **15,** 319–326 (June 2005).

22. Perrone, G. G., Tan, S.-X. & Dawes, I. W. Reactive oxygen species and yeast apoptosis. *Biochimica et Biophysica Acta (BBA) - Molecular Cell Research* **1783,** 1354–1368 (July 2008).

23. Halliwell, B. *Active Oxygen in Biochemistry* (2012).

24. Kanti Das, T., Wati, M. R. & Fatima-Shad, K. Oxidative Stress Gated by Fenton and Haber Weiss Reactions and Its Association With Alzheimer's Disease. *Arch Neurosci* **2** (Aug. 2014).

25. Ayer, A., Gourlay, C. W. & Dawes, I. W. Cellular redox homeostasis, reactive oxygen species and replicative ageing in *Saccharomyces cerevisiae. FEMS Yeast Research* **14,** 60–72 (Feb. 2014).

26. Cobley, J. N. Mechanisms of Mitochondrial ROS Production in Assisted Reproduction: The Known, the Unknown, and the Intriguing. *Antioxidants* **9,** 933 (Sept. 2020).

27. Dawes, I. W. & Perrone, G. G. Stress and ageing in yeast. *FEMS Yeast Research* **20,** foz085 (Feb. 2020).

28. Lu, H. *et al.* A consensus S. cerevisiae metabolic model Yeast8 and its ecosystem for comprehensively probing cellular metabolism. *Nature Communications* **10.** Number: 1 Publisher: Nature Publishing Group, 3586 (Aug. 2019).

29. Borgqvist, J., Welkenhuysen, N. & Cvijovic, M. Synergistic effects of repair, resilience and retention of damage determine the conditions for replicative ageing. *Sci Rep* **10,** 1556 (Dec. 2020).

30. Schnitzer, B., Borgqvist, J. & Cvijovic, M. The synergy of damage repair and retention promotes rejuvenation and prolongs healthy lifespans in cell lineages. *PLoS Comput Biol* **16,** e1008314 (Oct. 2020).
