# Supplementary material for: Multi-scale model suggests the trade-off between protein and ATP demand as a driver of metabolic changes during yeast replicative ageing
Source: PLoS Comput Biol. 2022 Jul 7;18(7):e1010261. doi: 10.1371/journal.pcbi.1010261 (PMC9295998; doi:10.1371/journal.pcbi.1010261)
Supplement: S2 Text — (PDF) [file pcbi.1010261.s003.pdf]

Multi-scale model suggests the trade-off between protein and ATP demand as a driver of metabolic changes during yeast replicative ageing
Supplementary text 2: Computational guidelines

Barbara Schnitzer [*1,2], Linnea Österberg [*3], Iro Skopa [1,2], Marija Cvijovic [1,2]

May 18, 2022

[*] Authors contributed equally
[1] Department of Mathematical Sciences, Chalmers University of Technology, Gothenburg, Sweden
[2] Department of Mathematical Sciences, University of Gothenburg, Gothenburg, Sweden
[3] Department of Biology and Biological Engineering, Chalmers University of Technology, Gothenburg, Sweden

Contents

1 Parameters **2**

2 Pseudo-code for integrated simulation **3**

1 Parameters

In the lifespan simulations several parameters were fixed, according to literature and chosen with the help simulation results.

module	description	parameter	value	unit	estimation/reference
ecFBA	inital protein content	P_{tot}	0.46	$g(gDW)^{-1}$	[1]
	fraction of included enzymes	f	0.1799		estimated with help of [2]
	enzyme saturation	σ	0.4592		adapted such that ecFBA replicates chemostat experiment [3]
	growth associated maintenance	GAM	*	$mmol(gDW)^{-1}$	[4, 5]
	maximal growth rate		0.35	h^{-1}	
	maximal acetate production		1.25	$mmol(gDW \cdot h)^{-1}$	[6]
	maximal glycerol production		1.2	$mmol(gDW \cdot h)^{-1}$	[6]
	maximal pyruvate production		0.1	$mmol(gDW \cdot h)^{-1}$	[6]
	maximal damage production via peroxynitrite		0.1	$mmol(gDW \cdot h)^{-1}$	
	maximal non-growth associated maintenance	$NGAM_{max}$	0.7	$mmol(gDW \cdot h)^{-1}$	[7]
	maximal superoxide production in cytosol		10^{-3}	$mmol(gDW \cdot h)^{-1}$	needed to allow flux through damage reactions in cytosol
ODE	size proportion	s	0.64		[8]
	retention factor	re	0.3		[8, 9]
integrated model	threshold for glucose availability	glc_c^{in}	3.2914	$mmol(gDW \cdot h)^{-1}$	[5]
	threshold for damage production	d_c	10^{-3}	$mmol(gDW \cdot h)^{-1}$	estimated with help of simulations
	threshold for Trx1/2	trx_c	$2 \cdot 10^{-9}$	$mmol(gDW)^{-1}$	estimated with help of simulations, equals two times the precision of the LP solver
	time step	δt	0.1	h	
	time steps between signalling and regulation	n_{delay}	5		
	flexibility in growth	γ	0.5		estimated to allow enough flexibility in enzyme reallocation during regulation

Table 1: Fixed parameters and conventions used in the paper.

* The GAM value is a coefficient in the stoichiometric matrix S and a function of the growth rate. It increases linearly from 18 to 30 $mmol(gDW)^{-1}$ for growth rates between 0 and 0.285 h^{-1} (respiration in chemostat setting), and thereafter decreases from 30 to 25 $mmol(gDW)^{-1}$ for growth rates between 0.285 and 0.4 h^{-1} (fermentation in chemostat setting).

2 Pseudo-code for integrated simulation

Algorithm 1 Sketch of integrated lifespan simulation

\# INITIALISATION ###

boolean ← initialise Boolean model from model files "species.txt" and "rules.txt"
targets ← get targets of transcription factors in the Boolean model from "TFtargets.txt"
ϵ ← set regulation factor

ecFBA ← initialise ecFBA model from model file "reducedEcYeast_XXX.mat" as a linear program
if necessary, manually curate *ecFBA* (e.g. updating bounds, additional constraints, σ, f)
define objective function of *ecFBA* to maximal growth

$P(0)$, $D(0)$, $M(0)$ ← set initial conditions of the ODE variables
s ← set size proportion
re ← set retention factor
f_0, r_0 ← set non-metabolic damage formation and repair rate
δt ← set time step

\# SIMULATION ##

$t \leftarrow 0$
while true **do**

    # UPDATE ecFBA PARAMETERS ##################################

    if deletion experiment **then**
        $e_{max,k} \leftarrow 0.0 \ \ \forall$ deleted enzymes k
    end if

    set preconditions for time-step by
    $P_{tot} \leftarrow P(t)$
    $e_{pool,max} \leftarrow f \cdot \sigma \cdot P_{tot}$
    $NGAM_{min} \leftarrow NGAM_{max} \frac{D(t)}{P(t)+D(t)}$

    # SOLVE FOR THE FIRST TIME ###############################

    solve parsimonious *ecFBA*
    if *ecFBA* == INFEASIBLE **then**
        break
    end if

    restrict growth rate with some flexibility by
    $g \leftarrow ecFBA.growthRate$
    $ecFBA.growthRate_{min} \leftarrow g \cdot (1 - \gamma)$

    $GAM \leftarrow GAM(g)$

Algorithm 1 Sketch of integrated lifespan simulation (continued)

\# REGULATE ACCORDING TO BOOLEAN SIGNALLING ##################

run *boolean* to get the logical steady state

find all transcription factors tf with $boolean.tf.active == true$
match with the ones included in *targets* to get those target enzymes that are regulated

calculate ranks using
$(rank)_i \leftarrow 0$ for all enzymes i in $ecFBA$
for all enzymes i **do**
    $(rank)_i \ += 1$ for each active tf that upregulates e_i
    $(rank)_i \ -= 1$ for each active tf that downregulates e_i
end for

and update bounds on enzymes usages accordingly
for all enzymes i with $(rank)_i \neq 0$ **do**
    $\Delta_i \leftarrow$ calculate range of enzyme usage e_i in $ecFBA$ that does not change the
        objective value (enzyme variability analysis)
    if $(rank)_i > 0$ **then**
        $e_{min,i} \ += \Delta_i \cdot \epsilon$
    else if $(rank)_i < 0$ **then**
        $e_{max,i} \ -= \Delta_i \cdot \epsilon$
    end if
end for

\# SOLVE THE REGULATED MODEL #############################

solve regulated parsimonious $ecFBA$
if $ecFBA ==$ INFEASIBLE **then**
    break
end if

if overexpression experiment **then**
    $e_{min/max,k} \leftarrow 1.5 \cdot e_k \ \ \forall$ overexpressed enzymes k
    solve updated regulated parsimonious $ecFBA$ once more
    if $ecFBA ==$ INFEASIBLE **then**
        break
    end if
end if

\# SOLVE ODE MODEL WITH ecFBA PARAMETERS ###################

$f_m \leftarrow \sum ecFBA.damageProduction$
$g \leftarrow ecFBA.growthRate$

solve system of ODEs for δt starting with current $M(t)$, $P(t)$ and $D(t)$
$\frac{\mathrm{d}M(t)}{\mathrm{d}t} = gM(t)$
$\frac{\mathrm{d}P(t)}{\mathrm{d}t} = -(f_m + f_0)P(t) + r_0 D(t)$
$\frac{\mathrm{d}D(t)}{\mathrm{d}t} = +(f_m + f_0)P(t) - r_0 D(t)$

Algorithm 1 Sketch of integrated lifespan simulation (continued)

98:
99: # CELL DIVISION ##
100:
101: cell division
102: **if** $M(t) \geq s^{-1}M(0)$ **then**
103: $M(t) \leftarrow M(0)$
104: $P(t) \leftarrow (1 - re)P(t)$
105: $D(t) \leftarrow (1 + re)D(t)$
106: **end if**
107:
108: # SAVE STEP (IF WANTED) ####################################
109:
110: save variables of interest (e.g. $M(t)$, $P(t)$, $D(t)$, current number of divisions, g, ...)
111:
112: # UPDATE ecFBA PARAMETERS ##################################
113:
114: trigger signalling with the solution fluxes and enzyme usages for next time step
115: $boolean.glucose \leftarrow ecFBA.glucoseFlux > glc_c^{in}$
116: $boolean.H2O2 \leftarrow ecFBA.damageProduction > d_c$
117: $boolean.trx \leftarrow ecFBA.trxUsage > trx_c$
118:
119: unconstrain $ecFBA.growthRate$, $\mathbf{e}_{min}$ and $\mathbf{e}_{max}$ again for next time step
120:
121: $t \leftarrow t + \delta t$
122:
123: **end while**
124:

One can add a time delay of the regulation by running line 50 with the Boolean model that was generated n_{delay} time steps ago instead of the one from the current time step. The updates in the Boolean model from line 114-117 are then only relevant in n_{delay} time steps.

References

1. Famili, I., Forster, J., Nielsen, J. & Palsson, B. O. Saccharomyces cerevisiae phenotypes can be predicted by using constraint-based analysis of a genome-scale reconstructed metabolic network. *Proceedings of the National Academy of Sciences* **100,** 13134–13139 (Nov. 2003).
2. Wang, M., Herrmann, C. J., Simonovic, M., Szklarczyk, D. & Mering, C. Version 4.0 of PaxDb: Protein abundance data, integrated across model organisms, tissues, and cell-lines. *Proteomics* **15,** 3163–3168 (Sept. 2015).
3. Van Hoek, P., Van Dijken, J. P. & Pronk, J. T. Effect of Specific Growth Rate on Fermentative Capacity of Baker's Yeast. *Appl Environ Microbiol* **64,** 4226–4233 (Nov. 1998).
4. Nilsson, A. & Nielsen, J. Metabolic Trade-offs in Yeast are Caused by F1F0-ATP synthase. *Scientific Reports* **6,** 22264 (Mar. 2016).
5. Österberg, L. *et al.* A novel yeast hybrid modeling framework integrating Boolean and enzyme-constrained networks enables exploration of the interplay between signaling and metabolism. *PLOS Computational Biology* **17** (ed Csikász-Nagy, A.) e1008891 (Apr. 2021).
6. Leupold, S. *et al.* Saccharomyces cerevisiae goes through distinct metabolic phases during its replicative lifespan. *eLife* **8,** e41046 (Apr. 2019).
7. Lu, H. *et al.* A consensus S. cerevisiae metabolic model Yeast8 and its ecosystem for comprehensively probing cellular metabolism. *Nature Communications* **10.** Number: 1 Publisher: Nature Publishing Group, 3586 (Aug. 2019).
8. Borgqvist, J., Welkenhuysen, N. & Cvijovic, M. Synergistic effects of repair, resilience and retention of damage determine the conditions for replicative ageing. *Sci Rep* **10,** 1556 (Dec. 2020).
9. Schnitzer, B., Borgqvist, J. & Cvijovic, M. The synergy of damage repair and retention promotes rejuvenation and prolongs healthy lifespans in cell lineages. *PLoS Comput Biol* **16,** e1008314 (Oct. 2020).
