# Supplementary material for: Multi-scale model suggests the trade-off between protein and ATP demand as a driver of metabolic changes during yeast replicative ageing
Source: PLoS Comput Biol. 2022 Jul 7;18(7):e1010261. doi: 10.1371/journal.pcbi.1010261 (PMC9295998; doi:10.1371/journal.pcbi.1010261)
Supplement: Attachment — Submitted filename: Plos yMSA rebuttal.pdf [file pcbi.1010261.s009.pdf]

Comments to the Authors:
Please note here if the review is uploaded as an attachment.

Reviewer #1: In this manuscript, a hybrid model developed in previous work by the authors describing the central carbon metabolism and nutrient signaling in S. cerevisiae is augmented by ROS/RNS related metabolic reactions and signaling and coupled with three ordinary differential equations depicting mass balances of fractions of intact and damaged proteins and cell biomass. The authors employed the developed model to provide a nice and insightful study of replicative yeast aging and quantify the extent of protein damage in cells.
The text is rather clear and well-structured, however some details have to be explained. Specific comments follow.

We would like to thank the reviewer for a very careful and thorough evaluation of the manuscript. We have received very much appreciated, constructive and valuable comments and suggestions. We carefully addressed all comments which lead to a revision of the manuscript and the supplementary information.

Line numbers here refer to the manuscript with tracked changes. The numbers are slightly shifted in comparison to the normal manuscript.

Comments:
1. The authors assume that the total protein fraction is constant over the lifespan of S. cerevisiae. It seems that this assumption is strong, knowing that protein fraction variation across growth rates in S. cerevisiae is 45-55% and that some other yeasts have even more pronounced variations over different growth rates. Considering that significant metabolic changes arise over successive replication, I think that it is important that the authors discuss this assumption. Are experimental data about the total protein fraction over replicative aging available in the literature? Can the authors comment on how sensitive this work's conclusions would be if this assumption does not hold?

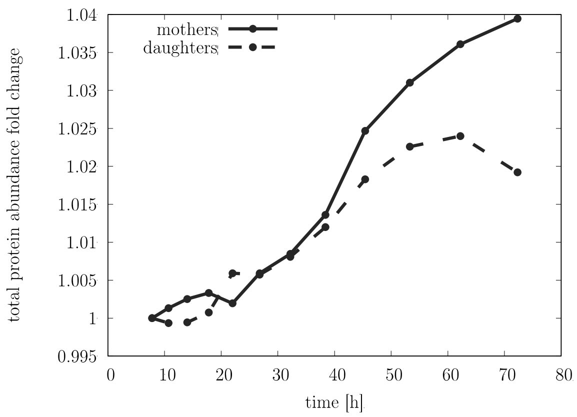

We agree that the assumption that the total protein fraction over time is constant is strong. However, we based it on data from Janssen et al., eLife 2015, that measured the relative abundance of a set of proteins in mothers and daughters over the replicative life of cells. Together, with absolute abundances from Ho et al., Cell Systems 2018, we could obtain an estimate of how much

the total protein abundance changes over time (age), and found that it is only an increase of about 4% (see figure above). We therefore decided to assume that the fraction is constant. We added a short justification also to the ms (line 433-436).

However, the fraction of **intact** proteins automatically varies over the life of a cell together with decreasing growth rates. Until cell death it decreases about a half (Fig 2B).

In general, it is noteworthy that our model displays in some sense an ideal cell, since we do not model any stochasticity and our model does not capture types of intrinsic and extrinsic variations. Imposing weaker or stronger (or also dynamic) constraints on the total fraction of proteins in the model would therefore just affect the timescale in the model, and metabolic switches or other type of changes as illustrated in Fig 2B appear later or earlier.

2. Following up on the previous remark, I could not find in the main manuscript or in the supplementary material (I did not check the code) is it there in the model a constraint that relates the intact and damaged proteins as a weighted sum of proteins in the biomass equation?

The biomass equation in the model is not affected by damage accumulation. We use the same equation as in the underlying FBA model from Österberg et al., PLoS Comp Bio 2021, and Nilsson et al., Scientific Reports 2016. It is only the upper bound on the enzyme pool that shrinks over time and forces different optimal flux distributions on the metabolic model.

3. Similarly, how exactly is fm(t) computed? I think that this information and information from 2. should be in the main manuscript as it will allow to readers understand the relationships between ecFBA and the ODEs better.

The damage formation rate fm(t) is an output of the metabolic (FBA) model. Consequently, the parameter is not computed, but is a result of the FBA optimization procedure. Fig1B shows details of the reactions we incorporated. Furthermore, on github (ModelFiles/ecFBA/Modified/DamageReactions) we provide the exact chemical reactions that were added to the FBA model.

In the ms, we now point the reader to github where relevant (lines 460-462, and section Simulation details) and added a small explanation (lines 426-428).

4. I could not find how much is ndelay?

The parameter ndelay is defined in the table in Text S2.

We assume that the delay between the moment a signal is received and when the metabolic model is affected is 30 minutes = 0.1h per timestep * 5 timesteps. In the Snf1 pathway, for instance, a similar time range was measured (Persson et al., FEMS Yeast Research 2022). In reality, the time of course depends on which protein it is, but for simplicity we set this to be a constant.

Similar to comment 1, we do moreover not expect qualitative differences if the delay was e.g. 20 or 40 minutes.

5. Table 1 – Perhaps I missed somewhere the definition, but what are "components"? Are these the metabolites and also the enzymes? In the table, when the size of the metabolic network is reported, the authors distinguish components and enzymes. Below, it seems that for yMSA the number of components involves the number of components from signaling and metabolic network but also the number of enzymes – this is confusing. Similarly, e.g., line 399 it is said "the ecFBA model was extended by 53 new reactions and 41 new components including 13 new enzymes" – how we should understand this statement? Please clarify.

We thank the reviewer for the comment. We agree that the formulations were misleading. We now updated and clarified Table 1, with enzymes as a separate category and focus on yMSA as a whole. To clarify, we added a short description of the types.

Components in the FBA model are the metabolites but also pseudo metabolites that are theoretical components that must be added when incorporating isoenzymes. In the Boolean model, they correspond to the signalling proteins and input metabolites. In the ODE, they describe the three states (M, P and D).

Enzymes are part of the metabolic model. The fluxes correspond to all chemical reactions that are included in the metabolic model. Additionally, enzyme-constrained FBA models typically contain pseudo reactions, which are also included here. Lastly, rules describe the interactions in the Boolean model.

We further adapted the formulation also in the Methods sections (lines 416-418).

6. Figure 2 – One can observe that glucose uptake is constant throughout the generations – ROS can inhibit multiple glycolytic enzymes, so, glucose uptake rate is likely to change over the generations. Please discuss this and acknowledge in the text. In the caption: "Stronger regulation increases the replicative lifespan and wildtype cells with more than 22 divisions cannot be achieved in this resolution if the regulation factor eps < 0.4" – is here 0.4 a typo? Should not be 0.04? Otherwise, I do not understand this statement from Figure 2, please clarify. Timescale in Figure 2B is in hours, please put in the figure. Fluxes are normalized with respect to what?

We thank the reviewer for spotting the mistake. We corrected the regulation factor to 0.04 in the ms.

In the model, the glucose uptake rate is not constant throughout the generations. In Figure 2B, we show the exchange fluxes normalised by the glucose uptake rate (see figure caption) since we focus on the changes in the metabolic pathways, observed via the exchange fluxes. In this case, glucose uptake is always -1. However, when investigating the absolute glucose uptake rate during ageing, it decreases along with the growth rate (and causes the signalling network to signal glucose depletion, Fig 2B).

We corrected the figure and clarified that the fluxes are normalised by the glucose uptake also in the axis label. To avoid confusion, we removed the dashed grey line for glucose, and acknowledge the decline in glucose uptake also in the text (line 139).

7. Can you please discuss in more detail on the nonmonotonic behavior of the curve around NGAM=0.25 mmol/gDW/h?

The non-monotonic behaviour for the damage at cell death around 0.25 is a result of the fact that we here plot the statistics of 29 parameter sets that could generate wildtype cells. Around that value, cells enter phase III and die in that phase. In the model, cell death occurs when the FBA part becomes infeasible. Sometimes, this can happen very abrupt after a cell division, but sometimes, cells can grow for some time before they die. This has a larger impact on phase II because growth rates are still higher, while in phase III there are typically not many divisions and very slow growth rates. In that way, the time between the last division and cell death are more sensitive to the parameter choice in phase II, explaining the variation and seemingly odd behaviour. Therefore, we do not believe that we can interpret too much in the high variation, but it is more an artefact of very diverse times between the last divisions and cell death.

We added some clarifications to the text, addressing this comment (lines 218-222).

8. Line 121, “The objective of the metabolic model is always maximal growth, but a certain flexibility is allowed” – can you be more precise/quantitative what do you mean by “a certain flexibility”?

We agree that the formulation is not concise and requires further explanation. The flexibility gamma defines how much the objective value, i.e. the maximal growth rate, is allowed to be violated after applying the regulation. This is necessary to avoid that the model becomes infeasible when applying the regulation. We removed the sentence in the Results section but clarified the Methods section in the ms (lines 398-399). All parameters, including gamma, are given in Text S2.

In our simulation, gamma is 50%, being a large margin. However, we observed that with decreasing functional enzymes the flexibility becomes more important, and using a large value helped us to avoid technical issues. We investigated the flexibility in more detail in a separate publication, that is linked to this one (https://doi.org/10.1101/2022.03.08.483444).

9. Line 377, “constrained” instead of “constraint”

We corrected the word in the ms.

Reviewer #2: I recommend this work for publication after a minor revision.

Summary
In this modeling work, the authors examine connections between replicative lifespan of budding yeast and two chemical species: Reactive Oxygen Species (ROS) and Reactive Nitrogen Species (RNS). An interesting aspect that the authors investigated is that both ROS and RNS are harmful to yeast but also are necessary and inevitable byproducts of metabolism. Specifically, the authors examined how yeast's metabolism changes as the cell's replicative age increases. To do so, the authors developed a multi-scale model that composed of a Boolean modeling of intracellular signaling, flux balance analysis (FBA) of metabolism, and a dynamical systems model of cell growth and an accumulation of damaged proteins. This model recapitulated known features of replicating aging

(e.g., avg. lifespan and subsequent cell division taking longer than the previous division). The modeling also revealed new features: how three distinct metabolic phases that the authors identified affect a yeast's replicative potential. Using this result, the authors proposed ways to control a yeast's lifespan.

We would like to thank the reviewer for a very careful and thorough evaluation of the manuscript. We have received very much appreciated, constructive and valuable comments and suggestions. We carefully addressed all comments which lead to a revision of the manuscript and the supplementary information.

Line numbers here refer to the manuscript with tracked changes. The numbers are slightly shifted in comparison to the normal manuscript.

Major points:

1. In their yMSA, it's unclear where the number of each components and rules listed in Table 1 came from. Importantly, it's unclear to me how randomly taking out some of these components or rules would qualitatively change the authors' conclusions. Is this a minimal model? I don't expect the authors to rerun their model and repeat all the work by randomly leaving out some of the modules. But I think some justification for choosing so and so many components and rules would be helpful to the readers.

We thank the reviewer for the comment. We agree that the formulations were misleading. We now updated and clarified Table 1. Furthermore, we added a short description of what is included as components, enzymes, rules and fluxes.

This is not a minimal model and the result of taking out components randomly depend on the component that is taken out. We wanted our model to reflect the current knowledge of the pathways included in the context of nutrient- and ROS signalling. The growth module is however a minimal model. It is an interesting research question to see what we can remove and still predict the WT behaviour; however, it was not something we focused on in this paper. We added a justification in the methods section (lines 418-422).

2. Why is it surprising that overexpressing Sod1, Sod2, Gpx3, and Glr1 completely or in phase II decrease the replicative lifespan? Give some explanation for this result.

It might be counterintuitive because Gpx3 and Glr1 are needed to remove H2O2 from the system, so one would assume that it is beneficial for the lifespan. At the same time, the overexpressions can force more flux through the electron transport chain and therefore enhance respiration, which can have a negative impact on growth and cell divisions. Yet, it is surprising that Trx1/2 and Trr1 manage to increase the lifespan when overexpressed in the same phase, even though they have a similar effect than Gpx3 and Glr1 in the model. This can be caused by many things, including different regulation or different enzyme efficiencies. However, this is pure speculation and needs further thorough study.

We removed the “surprisingly” in the text and tried to clarify the results including the correction of few mistakes (lines 289-294).

3. Related to 2: several results are given without an intuitive / biological explanation of why they make sense. I know that it's difficult to do so for complex pathways but I think the readers would be better served even if some speculation is given for the results.

We agree with the reviewer that in some cases we do not provide an extensive explanation for why we obtain certain results, in particular for specific gene knockouts and enzyme perturbations. This is because we work with a highly complex system and the explanations are often beyond intuition, and need a thorough computational analysis or experimental verification that was outside the scope of this work. While we tried our best to include as much experimental evidence as possible, we now also edited the ms again and extended explanations and speculations along with the suggestion of the reviewer (lines 289-294, 267-273, 290-294).

Reviewer #3: Building a multi-scale model of yeast replicative ageing (including an enzyme-constrained FBA model, a Boolean model of nutrient signalling pathways and dynamic model of protein damage accumulation and cell growth), Schnitzer et al aimed to investigate changes in the metabolism during replicative aging. Their model is unique due to the integration of three modules.

The model could reproduce close-to-experimental values for average replicative lifespan and generation times of wild-type yeast cells. The model also predicts features of replicative aging with distinct metabolic phases.

This is a timely work that aims to fill in an essential gap in quantitatively understanding aging and lifespan at the single-cell level. While I am overall positive, implementation of the following suggestions would be needed as they would further improve the impact of this work.

We would like to thank the reviewer for a very careful and thorough evaluation of the manuscript. We have received very much appreciated, constructive and valuable comments and suggestions. We carefully addressed all comments which lead to a revision of the manuscript and the supplementary information.

Line numbers here refer to the manuscript with tracked changes. The numbers are slightly shifted in comparison to the normal manuscript.

1. While the size of the individual modules of this multi-scale model is provided in a table, it would be good to also explain/inform the reader about which parameters have been empirically known and which have not. Also, any quantitative assessment of the robustness of the overall model should ideally be made.

In the table in Text S2, we provide a summary of all used parameters together with previous studies motivating the choice of the parameter.

The regulatory and the metabolic modules have been extensively studied before (Welkenhuysen et al., Frontiers in Physiology 2019, Nilsson et al., Scientific Reports 2016, Österberg et al., PLoS Comp Bio 2021). We therefore focused on the ODE part and the interfaces, where we assessed the robustness mostly via parameter explorations. The most sensitive part for the robustness of the whole model is the metabolic model that if infeasible induces cell death. We found that regulation (via regulation factor epsilon) can lead to overconstraining the metabolic model and inducing cell death too early, limiting the connection between the Boolean and the FBA model (Fig 3A). The

flexibility gamma is another measure to avoid the infeasibility of the model, that we investigated in the publication that is linked to this one (https://doi.org/10.1101/2022.03.08.483444).

We further simulated the effects of the ODE parameters f0 and r0 and tested their effect on the lifespan (Fig 2), but we could not observe any robustness issues.

It is noteworthy that our model displays in some sense an ideal cell, since we do not model any stochasticity and our model does not capture types of intrinsic and extrinsic variations. Consequently, the results we obtain are deterministic given an initial condition. Based on this and the studies we provided, we do not expect weird behaviour by changing parameters, but rather changes on the timescales.

2. As the authors also acknowledge, the phenotypic effects of some of the gene deletions cannot be reproduced by the model. It would be good if the authors at least tried to explain these shortcomings in a gene-to-gene basis (i.e. what changes in the model would make the model predict the phenotypic effect of gene x's absence?)

This in an interesting research question and would certainly be exciting to dive into. We partly covered this question in the Discussion section but did not pinpoint on specific gene knockouts. Here, we deal with a highly complex time-dependent network, such that such perturbations are beyond intuition and need thorough computational analysis, that was outside the scope of this work. Nonetheless, we tried to extend respective explanations in the text (lines 189-193).

3. Protein damage repair could be one type of NGAM, but could there be others? Potential other processes should be mentioned.

NGAM is not a very well-defined term, and it certainly encompasses other processes besides damage repair, since it traditionally refers to the substrate yield that is used for all other processes than growth. However, there is no consensus of what constitutes the NGAM. We dedicated a part in the discussion to this question and referred to several publications that discuss this question.

4. There are previously-published yeast-aging-focused or time-dynamic single-cell-modeling-focused papers that should have been cited due to their relevance to the different sections of this manuscript:

http://doi.org/10.1016/j.celrep.2019.07.082
http://doi.org/10.1186/s12859-019-2921-3
http://doi.org/10.1186/s12918-015-0240-5

We acknowledge the relevance of this work and added the references to the ms.

5. In the absence of direct experimental validations, the sections on NGAM and enzyme perturbations are not currently looking to be very strong. They are simulation results obtained from a model with lots of components/parameters without full in vivo values. This is not meant to be a major criticism; aging is a complex process and it is not surprising that it is taking a multiscale model to predict features to be experimentally tested. To improve the manuscript in these aspects, it would be good if the authors found as much relevant information from experimental literature as possible to validate bits and pieces of the predictions of the model. While this understandably cannot be comprehensive, it would help with the validation of the model.

We agree that it would have been great to provide with experimental verification for some of the hypothesis we have. We performed an extensive literature search already to back up as much as we could. The experimental verification of the hypothesis regarding the NGAM and exemplary enzyme perturbations is ongoing work. Nonetheless, we now edited the ms again and extended explanations, speculations and verifications along with the suggestion of the reviewer (lines 189-193, 267-273, 290-294, 433-436).
